# Prevalence and antibiotic resistance profile of *Listeria* spp. associated with seafoods from fish catchment areas in Kerala, India

**DOI:** 10.14202/vetworld.2021.777-783

**Published:** 2021-03-27

**Authors:** K. Vrinda Menon, B. Sunil, C. Latha

**Affiliations:** 1 Department of Veterinary Public Health, College of Veterinary and Animal Sciences, Kerala Veterinary and Animal Sciences University, Thrissur, Kerala, India; 2 Department of Veterinary Public Health, College of Veterinary and Animal Sciences, Veterinary and Animal Sciences University, Thrissur, Kerala, India; 3 Department of Veterinary Public Health, College of Veterinary and Animal Sciences, Veterinary and Animal Sciences University, Mannuthy, Thrissur, Kerala, India

**Keywords:** Antibiotic, Kerala, *Listeria*, seafoods, serotype

## Abstract

**Background and Aim:**

*Listeria monocytogenes* is a ubiquitous, intracellular pathogen which has been implicated as a cause of several foodborne outbreaks. This study aimed to generate information on the prevalence and antibiotic resistance profile of *Listeria* species isolated from seafood.

**Materials and Methods:**

A total of 400 samples of fresh fish, 100 samples of dry fish and 200 samples each of crustaceans and mollusks were collected from the fish catchment areas. All the samples were subjected to isolation and identification of *Listeria* spp. by two-step enrichment in UVM broth and plating on selective agar media (PALCAM) and then subjected to molecular characterization. *L. monocytogenes* isolates obtained during the study were subjected to serotyping by multiplex polymerase chain reaction. The isolates were also subjected to antibiotic sensitivity test.

**Results:**

The prevalence of *L. monocytogenes* in seafoods in the present study was 0.55%. The isolates of *L. monocytogenes* were found to possess all virulence genes, namely, *iap, hly*A*, act*A, *prf*A, *plc*A, and *inl*A. All the isolates belonged to serotype 4b. The occurrence of *Listeria innocua* was found to be more and was detected in 16.77% of seafood samples. Antibiotic sensitivity test revealed that all isolates were resistant to cefixime but were sensitive to almost all other commonly used antibiotics.

**Conclusion:**

The presence of *Listeria* spp. in raw seafood samples augments the need for implementation of good hygienic practices during the handling and processing of seafoods to safeguard the health of the consumers.

## Introduction

*Listeria monocytogenes* is one among the many causative agents of foodborne diseases in humans. The intracellular pathogen has been implicated in several outbreaks of foodborne diseases. The genus *Listeria* currently includes 17 recognized species of small rod-shaped Gram-positive bacteria [1]. The ubiquitous nature of *Listeria* spp. allows easy access of this pathogen to a variety of raw foods, including seafoods [2]. In spite of the wide distribution of the microorganism in the environment and relatively high frequency of isolation in foods, the incidence of listeriosis is low in the general population. The incidence of systemic listeriosis is much higher in individuals with compromised immune systems. The ingested organisms can breach endothelial and epithelial barriers of the infected host including the intestinal, blood-brain, and placental also [3]. According to the WHO, listeriosis is a relatively rare disease with 0.1-10 cases per 1 million people per year, depending on the countries and regions of the world. Despite the fact that the number of cases of listeriosis is low, the high rate of death associated with this infection makes it a significant public health concern. The antibiotic sensitivity profile of *Listeria* spp., however, varies with the use of drugs in human and veterinary medicine in different geographical areas. Inappropriate use of antibacterial drugs is the major cause of acquired resistance in *Listeria* species [4]. Multiple drug resistance in *L. monocytogenes* strains isolated from fish, raw and ready-to-eat seafood product samples has been reported [5]. Therefore, continuous focus on antibiotic-resistant *Listeria* isolates is essential to circumvent future risks to the human population.

The consumption of seafoods has increased globally because of its easy digestibility, high-quality protein content, and good source of lipids with high levels of unsaturated fatty acids [6]. The global proliferation of pathogens, especially *Listeria* spp. through seafood, is a major concern as it frequently triggers regulatory alerts in importing countries. Sporadic cases of foodborne listeriosis associated with seafoods have been reported across the globe for the past few decades. Extensive studies to assess the presence of *Listeria* spp., in the major fish catchment areas, have been very few in Kerala, India. With the advent of globalization of trade, ensuring the quality of seafood for exporting country like India becomes important for acceptability in the international market and for good economic returns [7].

This study aimed to generate information on the prevalence and antibiotic resistance profile of *Listeria* species isolated from seafoods.

## Materials and Methods

### Ethical approval

Ethical approval was not necessary for this study. However, samples were collected as per standard collection procedure.

### Study area and period

Seafood samples were collected from fish catchment area in coastal districts of Kerala from December 2013 to January 2015. The fish including dry fish, crustaceans, and mollusks were collected from Kozhikode (Puthiyappa harbor), Kollam (Neendakara), Thiruvananthapuram (Vizhinjam, Shangumugham), Alappuzha, and Thrissur (Chavakkad) districts in Kerala.

### Sampling

A total of 400 samples of marine fish, namely, sardine, mackerel, tuna, fin bream, anchovy, and milkfish were collected from the five coastal districts of Kerala. A total of 200 samples each of crustaceans (prawn and crabs) and mollusks (squid, mussels, and clams) were also collected from the areas under study. The fish, mollusks, and crustacean samples were collected soon after unloading from the boats in the harbor except in Alappuzha and Thrissur districts where the samples were collected after it was stored in ice. The dry fish samples stored at 25ºC (25 samples each from five districts) were collected from retail outlets near the harbors which mainly included two species of fish, namely, tongue sole and silver belly. The samples were collected in UV sterilized polyethylene bags and were brought to the laboratory in thermocol containers and immediately subjected to microbiological analysis.

### Isolation of *Listeria* species

The modification of the USDA [8] and FDA [9] protocols was used for the isolation of *Listeria* spp. It included a two-step enrichment of the sample in University of Vermont broth (UVM I) for 24 h at 37°C followed by inoculation in UVM II broth for 48 h at 37°C. The selective plating after enrichment was done in Polymyxin-Acriflavine-Lithium Chloride-Ceftazidime-Aesculin-Mannitol (PALCAM) (HiMedia) agar. The morphologically typical gray-green colonies with black sunken centers were verified by Gram’s staining and motility test and were also subjected to biochemical tests, that is, methyl red, Voges–Proskauer, sugar fermentation tests (rhamnose, mannitol, and xylose), catalase test, and Christie Atkins Munch Petersen (CAMP) test which are specific for *Listeria* spp. [10]. The positive isolates were further confirmed by molecular confirmation by polymerase chain reaction (PCR).

### Molecular confirmation of isolates

The DNA template for PCR was prepared by simple boiling and snap chilling method. PCR was standardized for the detection of genus-specific 16S rRNA followed by the standardization of six virulence genes which encode for phosphatidylinositol phosphatase C activity (*plcA*) (1484 bp), regulatory activity (*prfA*) (1060 bp), actin polymerization protein (*actA*) (839 bp), hemolysin activity (*hlyA*) (456 bp), p60 protein (*iap*) (131 bp), and internalin A protein (*inlA*) (800 bp) [11,12]. The standardization was done using *L. monocytogenes* standard strain MTCC 1143 obtained from IMTECH, Chandigarh. The suspected *Listeria innocua* isolates were confirmed by subjecting the isolates to *iap* gene. The genes were standardized using standard strain *L. innocua* (ATCC 33090). The *iap* gene encoding p60 protein was used as a target which is common to all members of the genus *Listeria*. The isolates of *L. innocua* showing weak hemolysis on blood agar plates were also subjected to *hlyA* gene to study their virulence.

### Serotyping of *L. monocytogenes* isolates

In this study, PCR was used to classify *L. monocytogenes* into different serogroups [13]. The multiplex PCR was performed using four sets of primers, namely, *lmo*0737, *lmo*1118, ORF2819, and ORF2110 with an amplification size of 906 bp, 690 bp, 471 bp, and 597 bp, respectively. This serogrouping was based on the fact that *L. monocytogenes* serotypes 1/2a, 1/2b, 1/2c, and 4b were responsible for 98% of documented listeriosis cases [14].

### Antibiotic susceptibility testing of the isolates

All *Listeria* isolates were subjected to antibiotic sensitivity test against 12 different antimicrobial agents by agar diffusion method in Mueller-Hinton AGAR [15]. *Listeria* isolates were tested against amoxicillin (10 mg), cefixime (10 mg), chloramphenicol (25 mg), furazolidone (100 mg), co-trimoxazole (25 mg), oxytetracycline (30 mg), erythromycin (15 mg), chlortetracycline (30 mg), streptomycin (10 mg), and nitrofurazone (100 mg), neomycin (10 mg), doxycycline hydrochloride (30 mg), and antibiotic discs (HiMedia, Mumbai). The clinical breakpoints for *Listeria* susceptibility testing were defined according to the Clinical and Laboratory Standard Institute [16] and the isolates were grouped as sensitive, intermediary sensitive, and resistant against each antibiotic.

### Statistical analysis

Data were analyzed statistically using software SPSS Version 21 (IBM, NY, USA).

## Results

### Occurrence of *Listeria* species in seafoods

**Figure-1:**
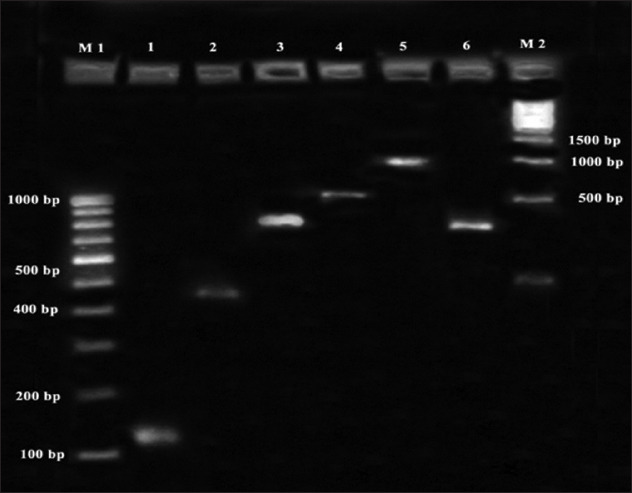
Polymerase chain reaction profile of *Listeria monocytogenes* isolate obtained with six virulence associated genes. Lane M: 100 bp ladder, lane 1: *iap* gene (131 bp), lane 2: *hly* A gene (456 bp), lane 3: *act* A gene (839 bp), lane 4: *prf* A gene (1060 bp), lane 5: *plc* A gene (1484 bp), lane 6: *Inl* A gene (~800 bp), lane M: 500 bp ladder.

Out of the 900 seafood samples screened, colonies from 156 samples showed characteristics of *Listeria* spp. in selective media which were later confirmed by biochemical tests. The results were further confirmed by the pathogenicity assay. Only isolates from five samples were positive for beta-hemolysis by CAMP test with *Staphylococcus aureus* and were confirmed as *L. monocytogenes*. *L. monocytogenes* isolates obtained were subjected to genus-specific 16S rRNA followed by the presence of six virulence genes. All the isolates of *L. monocytogenes* obtained revealed the presence of all six virulence genes (Figure-1). *L. monocytogenes* was isolated from dry fish (tongue sole), fish (Tuna), mollusks (mussels), and crustacean (prawns) samples. The remaining samples showing positivity in selective medium were found to be *L. innocua* when subjected to biochemical tests. The details of the samples found positive for *Listeria* spp. in different districts and from different species of seafoods are shown in Table-1.

**Table-1:** District-wise distribution of *Listeria innocua* in seafoods.

District	Number of samples positive for *Listeria* spp.				Total 900

	Fishes 400	Dry fish 100	Crustacean 200	Mollusks 200	
Kozhikode	21	2	4	8	35
Thrissur	14	1	3	2	20
Alappuzha	10	0	2	2	14
Kollam	20	1	35	8	64
Thiruvananthapuram	14	1	3	5	23
Total	78	4	47	22	156

**Figure-2:**
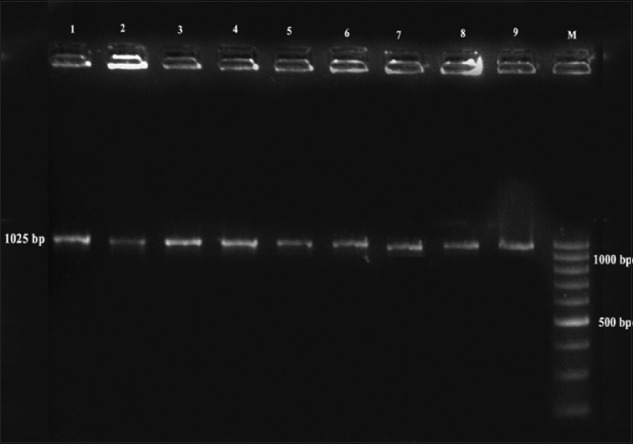
Polymerase chain reaction profile of *Listeria* innocua isolates from seafoods. Lane M: 100 bp ladder, lane 1-10: Isolates from seafoods.

**Figure-3:**
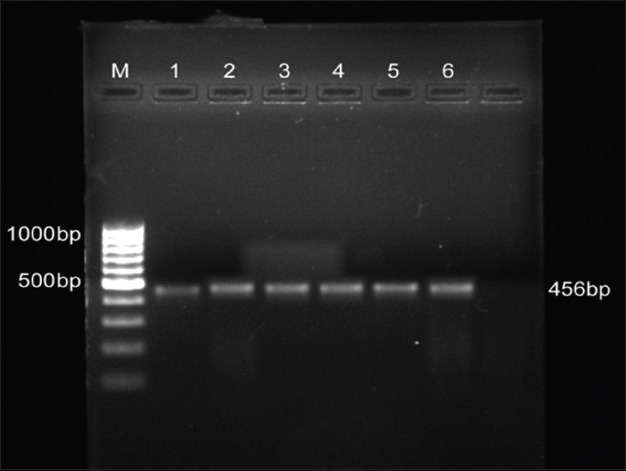
Polymerase chain reaction profile of *Listeria* *innocua* positive for *hly*A. Lane M: 100 bp ladder, lane 1: BD1, lane 2: P1, lane 3: P2, lane 4: H6, lane 5: 1143, Lane 6: H10.

*L. innocua* isolates were confirmed by subjecting the isolates to PCR targeting genus-specific 16S r RNA and *iap* gene. The occurrence of *L. innocua* in catchment areas of Kozhikode, Kollam, Thrissur, Thiruvananthapuram, and Alappuzha districts was 18.91, 34.59, 10.81, 12.43, and 7.56%, respectively. *L. innocua* isolates positive for *iap gene* are shown in Figure-2. Moreover, the prawn isolates obtained from Thrissur were found positive for the presence of *hlyA* gene (Figure-3). The maximum prevalence of *Listeria* spp. was observed in crustaceans followed by fishes and mollusks. The overall prevalence of *Listeria* spp. in seafoods was found to be 17.33%.

Statistical analysis of data using Chi-square multiple proportion test was done to know whether there was any significant difference between the prevalence of organism in different districts. This was followed by “Z” test, between the districts in which the organism was present. The results have shown that the presence of organism in Kollam was significantly higher compared to other districts (p<0.01).

### Serotyping of *L. monocytogenes* isolates

**Figure-4:**
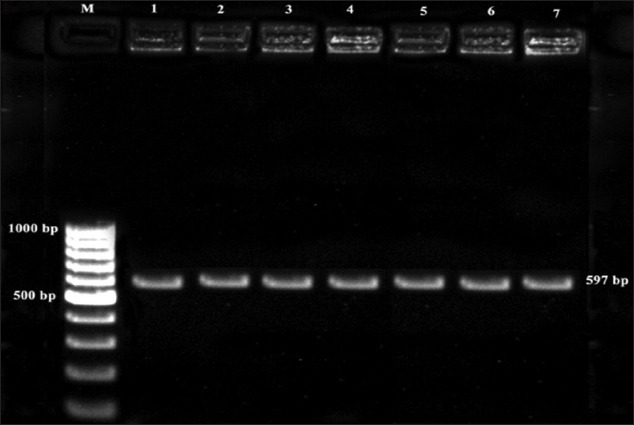
Multiplex PCR profile for serotyping of *L. monocytogenes* isolates. Lane M: 100 bp ladder, Lane1: Dry fish, Lane 2: Prawn, Lane 3: Mussels-1, Lane 4: Mussels-2, Lane 5: Fish Lane 6: Prawn 2, Lane 7: 1143.

*L. monocytogenes* isolates obtained during the study were subjected to serotyping using multiplex PCR with four sets of primers, namely, *lmo*0737, *lmo*1118, ORF2819, and ORF2110. This serogrouping was based on the fact that *L. monocytogenes* serotypes 1/2a, 1/2b, 1/2c, and 4b are responsible for 98% of documented listeriosis cases. The results indicated that all the isolates from fish, dry fish, mussels, and prawn samples revealed an amplification of 597 bp. Hence, it was concluded that the isolates belonged to Lineage I and the probable serotype was 4b, as shown in Figure-4.

### Antibiotic susceptibility testing of the isolates

The antibiotic sensitivity pattern of *Listeria* isolates from seafoods revealed that most of them were sensitive to the antibiotics under study. All isolates were resistant to cefixime. The isolates from dry fish showed intermediate resistance toward chlortetracycline, furazolidone, nitrofurazone, and cotrimoxazole but fresh fish isolates were sensitive to aforesaid antibiotics. All *L. innocua* isolates were sensitive to most of the antibiotics understudy but intermediate resistance was shown against nitrofurazone, cotrimoxazole, streptomycin, and amoxicillin. The maximum intermediate resistance against nitrofurazone (70.21%) was obtained from isolates obtained from crustaceans. *L. innocua* isolates obtained from mollusks also revealed resistance against cotrimoxazole (4.54%) and oxytetracycline (9.1%).

## Discussion

The present investigation revealed an overall prevalence of 0.55% and 16.77% of *L. monocytogenes* and *L. innocua*, respectively, in seafoods collected from catchment areas of five coastal districts of Kerala. The occurrence of *L. monocytogenes* in fish, dry fish, prawn, and mussels was found to be 0.25%, 2%, 0.5%, and 1%, respectively. The possible route of contamination may be attributed to the spread of the organism from the intestinal contents of fish to other tissues or through cross-contamination from contaminated surfaces, improper handling, and inappropriate transport. However, a higher prevalence of *L. monocytogenes* (2.7%) was observed in marine fish and fish products in Kerala [17] than in the present study. Higher prevalence of *L. monocytogenes* in fishes collected from Kerala, Iran, and Northeast India than the present study has also been recorded [17-19] with a prevalence of 1.66, 2.55, and 1.8%, respectively. Moreover, a higher prevalence of 6.32% in marine aquatic products in China was reported [16], which was higher than the present study. The variation in the results in different studies is related to differences in the rate of seafood contamination, isolation, and identification methodologies. The prevalence of *L. monocytogenes* in 2% of the dry fish samples in the present study was lower than the report of 5% in Kerala [20] but higher than the recent report in Kerala [17] where the organism could not be detected from dry fishes. The presence of the organism in dry fish in the present study could be attributed to the presence of the organism in fresh fish where the organism could have survived in the presence of less competitive bacteria, low water activity, and increased salinity. The isolation of *L. monocytogenes* in raw seafoods may pose a risk in kitchen through cross-contamination by handling cooked and raw foods, contamination from fish cleaning areas, and cutting board surface. Hence, avoidance of consumption of insufficiently cooked seafoods, especially by immunosuppressed individuals, is recommended.

*Listeria innocua* was the predominant species isolated from seafoods with a prevalence of 19.5%, 4%, 23.5%, and 11% from fish, dry fish, crustaceans, and mollusks, respectively. The increased occurrence of *L. innocua* in shellfishes may be attributed to their filter feeding habit which concentrates the microorganisms in their tissues. The prevalence of *L. innocua* (17.2%) reported in marine fishes and fish products [17] were in accordance with the present investigation. This finding is in agreement with some previous studies [1,5,20] who had reported a predominance of *L. innocua* in seafood samples procured from Goa, Cochin (Kerala), and Greece, respectively. The higher occurrence of *L. innocua* as compared to *L. monocytogenes* could be due to the fact that *L. innocua* has a shorter generation time than other *Listeria* spp. [11] and the recovery of *L. monocytogenes* using selective broth was lower when *L. innocua* was present. Moreover, *L. monocytogenes* and *L. innocua* share the same ecological niche and *L. innocua* could be used as an indicator for the presence of *L. monocytogenes* [21].

The occurrence of the organisms in seafoods suggests the need for environmental sanitation to control the organism in raw seafoods. In the present study, all *L. monocytogenes* isolates obtained and confirmed by culture techniques were found to be positive for the presence of all the six virulence genes. Similarly, the presence of all six virulence genes in *L. monocytogenes* isolates from clinical and environmental samples [22] and seafood samples [23] was also reported. The presence of six important virulence genes indicates that *L. monocytogenes* isolates in seafoods were capable of producing severe foodborne infections. The screening of other isolates based on PCR amplification of *iap* gene which encodes for p60 protein was confirmative of *L. innocua*. The presence of PCR product size of 1025 bp for *L. innocua* isolates from seafoods has been reported [24]. Weak hemolysis on blood agar and presence of *hly* A gene in *L. innocua* isolates from prawn samples was also observed in the present study which was in accordance with the reports which revealed the presence of atypical *L. innocua* strains (positive for *hly*A) among the isolates from food and environment by PCR [23]. These atypical *L. innocua* isolates could represent a stage in the evolution of *L. innocua* from a common ancestor of *L. monocytogenes* and might present a risk to human health [23]. Moreover, these atypical strains may constitute a reservoir of virulence genes transferable to other species of the same genus.

*L. monocytogenes* isolates obtained from seafoods were subjected to serotype identification by PCR. The results revealed that all the isolates belonged to serotype 4b which comes under lineage I. According to the previous studies, it has been observed that *L. monocytogenes* isolated from fish, fish products, and fish processing plants most often belonged to serotype 1/2a and 4b [25,26]. The results of earlier studies also revealed that more than 98% of infections were caused by serotypes 1/2a, 1/2b, 1/2c, and 4b [18,27]. Serotype 4b has been identified as the predominant serotype from raw fish [5], vegetable, soil clinical samples, and foods of animal origin [22]. However, the predominant serotype of *L. monocytogenes* from fish isolates in China belonged to serotype 1/2c [16] which differs from the findings of the present study and could be attributed to the regional differences and type of contamination. Serotype 4b has been implicated in majority of the foodborne outbreaks and has been implicated as the major cause of epidemics in humans and animals.

All *Listeria* isolates obtained from seafood ­samples showed sensitivity to erythromycin, chloramphenicol, doxycycline, chlortetracycline, and neomycin. The susceptibility of *L. monocytogenes* isolates to chloramphenicol and tetracycline was reported [28] which was in agreement with the present study. The isolates from dry fish showed intermediary resistance towards chlortetracycline, furazolidone and cotrimoxazole but fresh fish isolates were sensitive to aforesaid antibiotics. The efficiency of cotrimoxazole against *L. monocytogenes* as observed in the present study is in agreement with an earlier report [17]. A lower resistance to tetracycline and 100% susceptibility to streptomycin by *Listeria* isolates were observed [29] which were similar to the findings of the present study. The resistance of the isolates obtained from mollusks against cotrimoxazole and oxytetracycline was 4.54% and 9.1%, respectively. However, all other isolates were resistant to cefixime and sensitive to all other antibiotics under study. Similar findings on resistance to cephalosporin (ceftriaxone) against *Listeria* isolates obtained from foods were observed [30] which are in accordance with the present study. However, resistance to cefixime can be explained by the organism’s natural resistance to the antibiotic which is due to the minimal or non-existent affinity of listerial penicillin-binding protein 3 and 5 for cephalosporins [31]. All the isolates of *L. innocua* in the study were sensitive to chloramphenicol erythromycin doxycycline and neomycin. The reports of the sensitivity of the organism toward erythromycin and chloramphenicol [2] were in accordance with the present study. Mussels and fish isolates of *L. innocua* revealed intermediate resistance to nitrofurazone. The intermediate resistance to antibiotics is mainly due to the exchange of genetic material (antibiotic resistance genes) which may be due to persistence of *Listeria* in common environment [32]. Although the antibiotic resistance profiling of the isolates was not alarming, the chances of bacteria acquiring resistance to more drugs cannot be ruled out as the transfer of antibiotic resistance from other bacteria to *Listeria* has already been established.

## Conclusion

The prevalence of *Listeria* spp. from raw seafoods indicates a significant public health hazard. However, a high occurrence of *L. innocua* is an area of concern as the more pathogenic *L. monocytogenes* species share same ecological niche. Conscientious enforcement of sanitary conditions in fish handling areas, appropriate storage conditions, and personal hygiene practices would help to reduce the potential contamination of raw seafoods with *Listeria* spp. at the retail level and further reduce the bacterial load in the processed fish products. The antibiotic susceptibility pattern of the organism warrants the need for cautious use of these antimicrobials for use in veterinary and medical practice. Thus, there is a need for continual monitoring of the organism in for seafood exporting state like Kerala, as the organism frequently triggers regulatory alerts from importing countries. Moreover, the regular assessment and understanding of the antibiotic resistance pattern will help to effectively control the emergence of *Listeria* species in the region.

## Authors’ Contributions

KVM, BS, and CL: Designed the work. KVM collected the samples. KVM: Examined the samples in the research laboratory. All authors compiled, read, revised, and approved the final manuscript.
